# The effect of perennial and annual wheat forages, fed with or without lucerne, on the fatty acid profile and oxidative status of lamb meat

**DOI:** 10.1016/j.vas.2022.100230

**Published:** 2022-01-02

**Authors:** Benjamin W.B. Holman, Stephanie M. Fowler, Gordon Refshauge, Richard C. Hayes, Matthew T. Newell, Edward H. Clayton, Kristy L. Bailes, David L. Hopkins

**Affiliations:** a Centre for Red Meat and Sheep Development, NSW Department of Primary Industries, Cowra NSW 2794, Australia; b Cowra Agricultural Research and Advisory Station, NSW Department of Primary Industries, Cowra NSW 2794, Australia; c Wagga Wagga Agricultural Institute, NSW Department of Primary Industries, Wagga NSW 2678, Australia

**Keywords:** Grazing cereals, Sheep meat, Fatty acid profile, Vitamin E, TBARS, Alfalfa, Loin, Topside

## Abstract

The current study investigated the fatty acid profile and oxidative status of the meat from lambs that were fed a novel perennial wheat or a conventional annual wheat, either as a cereal monoculture or lucerne biculture. Twelve lambs were assigned to each of the four dietary treatments (48 lambs in total) and held within individual pens for the duration of the 28 day feeding study. Lambs were slaughtered and the *longissimus lumborum* (LL) and *semimembranosus* (SM) muscles analysed. The independent effect of wheat type on fatty acid concentrations was negligible. The concentration of long-chain saturated and omega-6 polyunsaturated fatty acids was higher when lucerne was included in the diet. Only monounsaturated fatty acids were affected by the interaction between wheat type and lucerne. The three-way interaction between wheat type, lucerne and muscle was only significant for the concentration of C12:0 and anteiso-C15:0. The concentration of thiobarbituric reactive substances and vitamin E was higher in meat from lambs fed a lucerne biculture, compared with those fed a cereal monoculture. Furthermore, and independent to dietary treatment, higher concentration of omega-3, omega-6 and other health claimable fatty acids were found in the SM, compared with the LL. This information will support industry adoption of novel perennial wheat polycultures and help producers to utilise it as a dual-purpose crop for the production of grain and/or sheep meat.

## 1 Introduction

There are economic and environmental advantages to grazing perennial cereal crops, especially when they are grown in combination with a legume (Ryan et al., 2018). Recently, a novel perennial wheat (breeding line 11,955, Hayes et al., 2012) was shown to support comparable growth rates and carcass properties to lambs fed a conventional, annual wheat (Newell et al., 2020). Further, consumer sensory panels and laboratory analyses have shown that the meat from lambs fed this same novel perennial wheat have ‘good everyday eating quality’ (Holman et al., 2021). In these reports, lamb performance and meat quality was not affected by the inclusion of lucerne into the forage mix (diet). The literature states that the diet of a grazing animal can affect the fatty acid profile and oxidative status of its meat (De Brito, Ponnampalam & Hopkins, 2017b; Sinclair, 2007). The effect of feeding a perennial wheat, as either a cereal monoculture or lucerne biculture, on these meat properties is unknown.

Although there is no research available to compare the effect of perennial and annual wheat types, past research has reported an effect of lucerne on the concentration of fatty acids in the meat of lambs. For example, Ponnampalam, Dunshea and Warner (2020) found that omega-3 (n-3) polyunsaturated fatty acid (PUFA) concentrations were enhanced in the meat of lambs supplemented with lucerne hay, when maintained on a low energy basal diet. Le et al. (2018) observed comparable α-linoleic acid (ALA) and n-3 PUFA concentrations in the meat of lambs grazing cocksfoot pastures or lucerne. The concentration of ALA, n-3 and total PUFA, in this previous study, was also found to be higher in the meat of lambs grazing lucerne, when compared with a ryegrass monoculture. Ponnampalam et al. (2017) compared the fatty acid profile of crossbred lambs grazing lucerne, annual ryegrass/subclover, and annual ryegrass pastures. This study found that the meat of lambs grazing lucerne had higher ALA, n-3 and total PUFA concentrations than the meat from lambs grazing a ryegrass monoculture (Ponnampalam et al., 2017). Frank et al. (2016) concluded that the selection of ryegrass, lucerne or brassica cultivars (*cv*. Titan and *cv*. Greenland) will impact on the fatty acid profile and oxidative stability of the meat of grazing lambs. Collectively, these studies demonstrate an opportunity for farmers to select production systems that enhance the concentration of health claimable PUFA in the meat of grazing lambs. These include ALA, linoleic acid, eicosapentaenoic acid (EPA) and docosahexaenoic acid (DHA), albeit the recommended daily dosage for humans differs between authorities and will depend on a consumer's physiological status (Ponnampalam, Sinclair & Holman, 2021). The stability of these health claimable PUFA over the interval between slaughter and consumption is also a function of the antioxidant capacity of the meat (Ponnampalam et al., 2014b).

The current study aimed to examine the effect of annual and perennial wheat, fed either as a monoculture or lucerne biculture, on the fatty acid profile, thiobarbituric reactive substances (TBARS), and vitamin E concentrations of meat.

## 2 Materials and methods

The current study adhered to the Australian Code of Practice for the Use of Animals for Scientific Purposes (National Health & Medical Research Council, 2013). Animal ethics approval was granted by the Animal Ethics Committee of the NSW Department of Primary Industries (ORA18/21/022).

### 2.1 Design

This study follows on from Newell et al. (2020) and Holman et al. (2021), which detail the feeding study and its effect on feed intake, animal performance, carcass, meat quality and sensory properties. Briefly, a stratified randomised block design was used to compare four dietary treatments, using a total of 48 lambs that were housed within individual pens (area: 3 × 12 m), allowing for 12 lambs (replicates) per dietary treatment. The allocation of lambs to a treatment was stratified by initial liveweight. The feeding study continued for 28-days and included a 7-day adjustment period. The dietary treatments were the forage of two cereal (wheat) types, perennial wheat (line 11,955) and annual wheat (cv. EGA Wedgetail). These were cut daily and delivered fresh to the lambs in combination with (+L) or without (-L) lucerne (cv. Titan 9). Details of the dietary treatments and average chemical composition are shown in Table 1.

**Table 1:** Summary data of the forage quality and concentration of selected fatty acid is shown for each of the wheat type and for lucerne. Mean ± standard deviation values are shown.^a^.

	PW	W	L
Forage quality^b^			
Dry matter,%	21.8 ± 1.9	22.6 ± 1.7	29.4 ± 3.3
Crude protein,%DM	24.3 ± 2.0	26.2 ± 2.3	18.9 ± 1.6
Neutral detergent fibre,%DM	44.8 ± 2.9	41.8 ± 2.0	34.3 ± 4.3
Acid detergent fibre,%DM	21.6 ± 1.4	19.7 ± 1.1	24.5 ± 3.2
Water soluble carbohydrate%DM	10.0 ± 1.9	13.9 ± 2.0	7.1 ± 1.3
Metabolisable energy, MJ/kg DM	12.0 ± 0.5	12.3 ± 0.3	10.6 ± 0.6
Fatty acid, mg/100 g fresh wt.			
C12:0	0.38 ± 0.02	0.39 ± 0.03	0.37 ± 0.05
C14:0	0.06 ± 0.01	0.06 ± 0.01	0.23 ± 0.04
C16:0	2.52 ± 0.16	2.61 ± 0.08	3.02 ± 0.14
C17:1n-7	5.12 ± 0.83	3.71 ± 0.85	0.03 ± 0.01
C18:0	0.40 ± 0.03	0.39 ± 0.04	0.53 ± 0.03
C18:1n-7	0.07 ± 0.01	0.06 ± 0.01	0.07 ± 0.01
C18:1n-9	0.26 ± 0.03	0.27 ± 0.03	0.27 ± 0.02
C18:2n-6	2.08 ± 0.20	1.86 ± 0.16	2.63 ± 0.33
C18:3n-3	13.94 ± 2.16	15.42 ± 1.47	8.64 ± 0.99
C20:0	0.17 ± 0.01	0.11 ± 0.01	0.19 ± 0.01
C20:1n-9	0.02 ± < 0.01	0.01 ± < 0.01	0.01 ± < 0.01
C22:0	0.19 ± 0.01	0.19 ± 0.02	0.23 ± 0.03
∑n-3	14.01 ± 2.17	15.50 ± 1.48	8.79 ± 1.0
∑n-6	2.17 ± 0.20	1.95 ± 0.16	2.95 ± 0.32
n-6:n-3 ratio	0.16 ± 0.03	0.13 ± 0.02	0.34 ± 0.04
∑SFA	4.09 ± 0.22	4.13 ± 0.16	5.42 ± 0.15
∑MUFA	7.15 ± 1.02	6.76 ± 1.26	1.30 ± 0.24
∑PUFA	16.18 ± 2.21	17.45 ± 1.34	11.73 ± 1.17

a Abbreviations include perennial wheat (PW); annual wheat (W); lucerne (L); dry matter (DM); sum of C18:3n-3, C20:3n-3, C20:4n-3, C20:5n-3, C20:5n-3 and C22:6n-3 (∑n-3); sum of C18:2n-6, C18:3n-6, C20:2n-6, C20:3n-6, C20:4n-6, C22:4n-6 and C22:5n-6 (∑n-6); sum of C10:0, C12:0, C14:0, C15:0, C16:0, C17:0, C18:0, C20:0, C22:0, C23:0 and C24:0 (∑SFA); sum of C14:1n-5, C15:1n-5, C16:1n-7t, C16:1n-7, C17:1n-7, C18:1n-9t, C18:1n-7t, C18:1n-9, C18:1n-7, C20:1n-15, C20:1n-9, C22:1n-9 and C24:1n-9 (∑MUFA); sum of C20:3n-9, ∑n-3 and ∑n-6 (∑PUFA).

b First published in Newell et al. (2020).

At the completion of the feeding study, all lambs were slaughtered, as a single flock. Medium voltage electrical stimulation was applied before the carcasses were trimmed and dressed, in accordance with normal industry practice. At 24 h *post-mortem* and from the left-side of each carcass, the *longissimus lumborum* (LL) and *semimembranosus* (SM) muscles were removed. These were vacuum packaged and aged for 5-days under refrigeration (mean ± standard deviation: 2.7 ± 0.5 °C). At this point, the muscles were sectioned, samples were removed and frozen at −80 °C until their analysis.

### 2.2 Fatty acid concentration

Samples of 25 g were freeze-dried at −50 °C (ScanVac Cool Safe, LaboGene Ltd., DEN) and then ground using a sample mill (model 1095, Knifetech, FOS Pacific Ltd., AUS). The fatty acid concentrations of the freeze-dried samples were determined using the modified one-step method of Clayton et al. (2012). First, 10 mg of sample were combined with 2 mL of methanol:toluene (4:1 v/v) and 10 μg/mL of each internal standard (C13:0 and C19:0). Fatty acids were methylated with the addition of 200 μL of acetyl chloride and the subsequent incubation at 100 °C for 60 min. Once cooled, 5 mL of 6% potassium carbonate solution was added to each sample. Centrifugation at 1500 × *g* for 10 min was then used to separate the upper toluene supernatant phase, which was transferred into a 2 mL glass vial fitted with a Teflon lined screw-cap lid.

Individual fatty acid methyl esters (FAME) were quantified using an Agilent 7890A GC fitted with a dual BPX70 capillary column (30 m × 0.25 mm i.d. and 0.25 μm film thickness, SGE Analytical Science, AUS) and a dual flame ionisation detector (FIDs). Helium was used as the carrier gas having a split ratio of 10:1, total flow rate of 12.4 mL per min, and a column flow rate of 0.9 mL per min. The inlet temperature was 250 °C, pressure was 107.8 kPa, and injection volume was 2.5 μL into a focus inlet liner (4 mm i.d., no. 092,002, SGE Analytical Science, AUS). The oven temperature was held at 150 °C for 30 s, before being increased by 10 °C per min up to 180 °C; then by 1.5 °C per min up to 220 °C; and lastly by 30 °C per min up to 260 °C. This final temperature was held for 5 min to result in a total run time of 36.5 min. FID temperature was 280 °C, its gas flow rate for helium was 35 mL per minute, instrument air was 350 mL per min, and nitrogen make-up gas was 30 mL per min. FAME peaks were identified against the retention times of commercial standards and published data (Clayton et al., 2012; Or-Rashid, Fisher, Karrow, Al Zahal & McBride, 2010). FAME concentrations were calculated against a 3-point standard curve. Cis- and trans-double bond geometries, and conjugated linoleic acids (CLA) are described. Concentrations of t11–18:1 and t10–18:1 were co-eluted and therefore have not been reported. Data were reported as mg per 100 g fresh (wet) sample weight. The abbreviations used to define summative fatty acid results are shown in Tables 1 and 2.

**Table 2:** The effect of wheat type, lucerne, muscle and their two- and three-way interactions on the fatty acid concentrations of the meat from experimental lambs. The significant effects are shown in bold (*P* < 0.05). The covariate effect of intramuscular fat content is included.^a^.

Fatty acid	Wheat type	Lucerne	Wheat type × Lucerne	Muscle	Wheat type × Muscle	Lucerne × Muscle	Wheat type × Lucerne × Muscle	Covariate
∑SFA	0.192	0.499	0.347	**0.001**	0.491	0.097	0.087	**< 0.001**
C10:0	0.098	0.631	0.889	0.340	0.600	0.153	0.279	**0.017**
C12:0	0.495	0.374	0.390	**< 0.001**	0.751	0.072	**0.028**	**< 0.001**
C14:0	0.557	0.418	0.533	0.828	0.387	0.094	0.075	**< 0.001**
C15:0	0.177	0.915	0.929	**< 0.001**	0.399	0.123	0.051	**< 0.001**
C16:0	0.236	0.200	0.443	**< 0.001**	0.476	0.122	0.103	**< 0.001**
C17:0	0.274	0.944	0.410	0.882	0.508	0.082	0.069	**< 0.001**
C18:0	0.386	0.845	0.482	**< 0.001**	0.528	0.082	0.084	**< 0.001**
C20:0	0.363	0.491	0.249	0.308	0.660	0.183	0.199	**< 0.001**
C22:0	0.924	**0.026**	0.150	0.228	0.99	0.670	0.201	0.443
C23:0	0.653	**< 0.001**	0.159	0.140	0.117	0.715	0.571	0.973
C24:0	0.901	**0.001**	0.121	0.884	0.711	0.734	0.173	0.913
∑BCFA	0.164	0.516	0.901	**< 0.001**	0.355	0.230	**0.043**	**< 0.001**
anteiso-C15:0	0.065	0.352	0.855	**< 0.001**	0.308	0.283	**0.047**	**< 0.001**
iso-C15:0	**0.046**	0.915	0.650	0.612	0.418	0.060	0.065	**< 0.001**
anteiso-C17:0	0.245	0.074	0.769	0.327	0.650	0.596	0.266	0.106
iso-C17:0	0.715	0.430	0.457	**< 0.001**	0.532	0.871	0.281	**0.005**
∑MUFA	0.742	**0.034**	**0.034**	0.173	0.374	0.173	0.116	**< 0.001**
C14:1n-5	0.500	0.515	0.779	**< 0.001**	0.105	0.277	0.114	**< 0.001**
C16:1n-7t	0.073	**< 0.001**	0.486	**<0.001**	0.308	0.443	0167	**< 0.001**
C17:1n-7	0.890	0.364	0.137	0.085	0.284	0.259	0.223	**< 0.001**
C18:1n-7	0.325	0.723	**0.049**	**< 0.001**	0.416	0.296	0.160	**< 0.001**
C18:1n-7t	0.418	0.103	0.323	**< 0.001**	0.572	0.586	0.889	0.880
C18:1n-9	0.887	**0.024**	**0.033**	0.338	0.404	0.136	0.109	**< 0.001**
C18:1n-9t	0.523	0.342	0.964	**< 0.001**	0.239	0.305	0.675	0.243
C20:1n-9	0.784	0.514	0.669	**< 0.001**	0.468	0.350	0.194	0.813
C20:1n-15	0.600	0.244	0.213	**< 0.001**	0.726	0.700	0.059	**0.033**
C22:1n-9	0.759	0.676	**0.015**	**0.012**	0.729	0.948	0.662	0.073
C24:1n-9	0.414	**0.037**	0.181	0.085	0.737	0.525	0.108	0.355
∑PUFA	0.341	**0.006**	0.527	**< 0.001**	0.295	0.371	0.390	0.723
C16:3n-4	0.713	0.941	0.136	0.133	0.235	0.319	0.096	0.154
C20:3n-9	0.512	0.329	0.903	**< 0.001**	0.307	0.415	0.855	0.081
∑n-3	0.623	0.082	0.971	**< 0.001**	0.355	0.578	0.280	0.169
C18:3n-3	0.430	0.150	0.768	**< 0.001**	0.327	0.778	0.286	**< 0.001**
C20:3n-3	0.161	0.204	0.914	**< 0.001**	0.948	0.513	0.119	0.478
C20:4n-3	0.640	**0.026**	0.362	**< 0.001**	0.142	0.088	0.313	0.704
C20:5n-3	0.892	0.155	0.574	**< 0.001**	0.490	0.174	0.785	**0.004**
C22:5n-3	0.778	0.062	0.672	**< 0.001**	0.418	0.185	0.224	0.235
C22:6n-3	0.995	0.388	0.726	**< 0.001**	0.801	0.273	0.608	**0.016**
∑n-6	0.206	0.002	0.284	**< 0.001**	0.293	0.268	0.495	0.603
C18:2n-6	0.076	**0.004**	0.314	**< 0.001**	0.268	0.419	0.439	0.667
C18:3n-6	0.577	0.830	0.399	**< 0.001**	0.799	**0.028**	0.088	0.536
C20:2n-6	**0.010**	0.473	0.526	0.159	0.107	0.115	0.202	**0.039**
C20:3n-6	0.132	**0.006**	0.252	**< 0.001**	0.382	0.093	0.715	0.125
C20:4n-6	0.867	**0.009**	0.328	**< 0.001**	0.577	**0.021**	0.945	**0.002**
C22:4n-6	0.487	**0.026**	0.763	**< 0.001**	0.450	**0.035**	0.470	0.137
C22:5n-6	0.939	0.139	0.872	**< 0.001**	0.343	0.163	0.475	0.107
n-6:n-3	0.308	**0.022**	0.143	**< 0.001**	0.778	0.922	0.781	0.522
∑CLA	**0.032**	0.390	0.934	**< 0.001**	0.515	0.366	0.219	**< 0.001**
c9t11CLA	**0.030**	0.386	0.800	**< 0.001**	0.791	0.448	0.336	**< 0.001**
t10c12CLA	0.062	0.444	0.729	0.862	0.199	0.344	0.152	**< 0.001**
∑Health claimable	0.920	0.141	0.785	**< 0.001**	0.672	0.176	0.713	**0.004**

a Abbreviations include those presented in the footnote of Table 1; sum of anteisoC15:0, isoC15:0, isoC17:0 and anteisoC17:0 (∑BCFA); sum of c9,t11CLA and t10,c12CLA (∑CLA); ratio of ∑n-6 to ∑n-3 (n-6:n-3); and sum of C20:5n-3 and C22:6n-3 (∑Health claimable).

### 2.3 Thiobarbituric acid reactive substances

Using the method of Holman, Bailes, Kerr and Hopkins (2019), 100 mg samples were homogenised with 0.5 mL of radio-immunoprecipitation assay buffer (no. 10,010,263, Cayman Chemical Company Ltd., USA). These were centrifuged and the supernatant tested in accordance with the thiobarbituric acid reactive substances (TBARS) assay kit colorimetric protocol (no. 700,870, Cayman Chemical Company Ltd., USA). A benchtop spectrophotometer (FLUOstar OPTIMA, BMG Labtechnologies, AUS) set to measure absorbence at 540 nm was used to calculate TBARS concentrations as mg malondialdehyde (MDA) per kg fresh (wet) sample weight.

### 2.4 Vitamin E

At a commercial laboratory (method NTM-31, DPRID Diagnostics and Laboratory Services, AUS), 1 g freeze-dried samples were homogenised with 10 mL of 6% pyrogallol and 1 mL of 60% potassium hydroxide (McMurray, Blanchflower & Rice, 1980). Samples were incubated for 30 min at 70 °C, cooled and then extracted with 5 mL of water and 20 mL of hexane. A 5 mL aliquot of the hexane layer, first evaporated under nitrogen gas, was reconstituted in 0.5 mL of methanol and analysed using an Agilent high performance liquid chromatograph (1260) fitted with a Zorbax SB-C18 column (3.5 μm i.d., 3 mm × 150 mm) for chromatographic separation. Vitamin E (α-tocopherol) concentrations were compared against a standard curve using fluorescence detection and a benchtop spectrophotometer set to measure emission at 330 nm and excitation at 296 nm. Data were transformed and reported as mg vitamin E per kg of fresh (wet) sample.

### 2.5 Statistical analysis

Data were analysed in Genstat (20th Edition, VSN International Ltd., www.vsni.co.uk) using analysis of variance (ANOVA) models. The main effects of wheat type (perennial and annual), lucerne (+*L* and -L), muscle (LL and SM), and their two- and three-way interactions were fitted as fixed terms. Animal was fitted as a random term. For the analysis of fatty acid data, intramuscular fat concentration was fitted into the model as a covariate. The intramuscular fat data was sourced from Holman et al. (2021). Differences between means were significant when *P* < 0.05.

## 3 Results

### 3.1 Fatty acid concentration

The effect of wheat type, lucerne, muscle type, and their interactions on the concentration of fatty acids in lamb meat is shown in Table 2. The concentrations of iso-C15:0, C20:2n-6, c9t11CLA and ∑CLA were found to be significantly higher in the meat of lambs fed perennial wheat (Table 3). The latter two results are connected as c9t11CLA was used in the calculation of ∑CLA.

**Table 3:** The fatty acid concentration of the meat from experimental lambs as per the independent effect of wheat type, lucerne and muscle. Means with different superscripts (shown in bold) were significantly different (*P* < 0.05). Mean and the standard error of the mean are shown.^a^

Fatty acid, mg/100 g	Wheat type			Lucerne			Muscle		
	PW	W	SEM	-L	+L	SEM	LL	SM	SEM
∑SFA	1270.5	1237.8	24.5	1262.7	1245.6	24.7	1301.5^a^	1206.7^b^	27.7
C10:0	19.6	19.0	0.3	19.2	19.4	0.3	19.4	19.2	0.3
C12:0	3.5	3.4	0.2	3.5	3.3	0.2	3.2^b^	3.6^a^	0.1
C14:0	63.8	62.0	2.4	63.8	61.8	2.4	62.9	62.6	1.6
C15:0	11.7	11.2	0.4	11.5	11.5	0.4	10.7^b^	12.3^a^	0.3
C16:0	655.0	638.2	13.8	655.7	637.5	13.9	671.5^a^	621.8^b^	14.2
C17:0	32.8	31.9	0.8	32.3	32.4	0.8	32.3	32.4	0.7
C18:0	478.0	465.8	14.0	470.6	473.3	14.1	495.3^a^	448.5^b^	11.0
C20:0	2.5	2.4	0.1	2.4	2.5	0.1	2.5	2.4	0.1
C22:0	1.1	1.1	< 0.1	1.1^b^	1.1^a^	< 0.1	1.1	1.1	< 0.1
C23:0	1.4	1.4	< 0.1	1.3^b^	1.5^a^	< 0.1	0.4	1.4	< 0.1
C24:0	1.4	1.4	< 0.1	1.3^b^	1.4^a^	< 0.1	1.4	1.4	< 0.1
∑BCFA	20.4	19.6	0.5	19.8	20.2	0.5	18.8^b^	21.2^a^	0.4
anteiso-C15:0	5.3	4.9	0.2	5.0	5.2	0.2	4.8^b^	5.4^a^	0.1
iso-C15:0	3.6^a^	3.3^b^	0.1	3.4	3.4	0.1	3.4	3.5	0.1
anteiso-C17:0	2.5	2.6	0.1	2.5	2.7	0.1	2.5	2.6	0.1
iso-C17:0	0.9	0.9	< 0.1	0.9	0.8	< 0.1	0.8^b^	0.9^a^	< 0.1
∑MUFA	1286.6	1276.2	29.3	1313.9^a^	1248.9^b^	29.5	1263.0	1299.7	25.9
C14:1n-5	2.4	2.3	0.2	2.4	2.3	0.2	2.1^b^	2.6^a^	0.1
C16:1n-7t	6.5	6.1	0.2	5.8^b^	6.7^a^	0.2	5.9^b^	6.7^a^	0.1
C17:1n-7	11.3	11.3	0.3	11.4	11.2	0.3	11.2	11.4	0.1
C18:1n-7	28.6	28.0	0.5	28.4	28.2	0.5	26.6	29.9	0.4
C18:1n-7t	38.0	34.6	4.3	32.7	39.9	4.3	13.7^b^	58.9^a^	4.2
C18:1n-9	1128.8	1123.8	29.6	1161.4^a^	1091.3^b^	29.7	1137.2	1115.4	23.3
C18:1n-9t	6.9	7.1	0.3	6.9	7.2	0.3	6.4^b^	7.6^a^	0.2
C20:1n-9	1.5	1.5	0.2	1.5	1.6	0.2	0.7^b^	2.4^a^	0.2
C20:1n-15	1.3	1.3	0.1	1.3	1.3	0.1	1.0^b^	1.6^a^	0.1
C22:1n-9	0.4	0.5	< 0.1	0.5	0.4	< 0.1	0.4^b^	0.5^a^	< 0.1
C24:1n-9	1.2	1.2	< 0.1	1.2^a^	1.1^b^	< 0.1	1.2	1.1	< 0.1
∑PUFA	267.8	263.1	5.0	258.2^b^	272.7^a^	5.0	242.4^b^	288.5^a^	2.2
C16:3n-4	0.5	0.5	< 0.1	0.5	0.5	< 0.1	0.5	0.5	< 0.1
C20:3n-9	9.1	9.4	0.5	9.0	9.5	0.5	8.6^b^	9.8^a^	0.1
∑n-3	114.8	113.6	2.5	112.0	116.5	2.5	105.6^b^	122.8^a^	0.9
C18:3n-3	58.3	57.2	1.4	56.7	58.7	1.4	54.2^b^	61.2^a^	0.6
C20:3n-3	1.3	1.3	0.1	1.3	1.3	0.1	1.2^b^	1.4^a^	< 0.1
C20:4n-3	2.1	2.1	0.1	2.0^b^	2.2^a^	0.1	2.0^b^	2.2^a^	< 0.1
C20:5n-3	22.7	22.8	0.7	22.3	23.2	0.7	20.5^b^	25.0^a^	0.2
C22:5n-3	21.9	21.8	0.5	21.4	22.3	0.5	20.3^b^	23.4^a^	0.2
C22:6n-3	8.6	8.6	0.4	8.4	8.7	0.4	7.6^b^	9.5^a^	0.1
∑n-6	140.2	136.6	2.9	133.7	143.2	2.9	124.8^b^	152.1^a^	1.3
C18:2n-6	93.1	89.5	2.0	88.2^b^	94.3^a^	2.0	82.1^b^	100.4^a^	0.9
C18:3n-6	2.0	2.0	0.1	2.0	2.0	0.1	1.9^b^	2.1^a^	0.1
C20:2n-6	1.1^a^	1.0^b^	< 0.1	1.1	1.1	< 0.1	1.1	1.1	< 0.1
C20:3n-6	4.2	4.0	0.1	4.0^b^	4.3^b^	0.1	3.9^b^	4.4^a^	0.1
C20:4n-6	32.9	32.7	1.0	31.4^b^	34.1^a^	1.0	29.2^b^	36.4^a^	0.3
C22:4n-6	1.6	1.6	0.1	1.5^b^	1.6^a^	0.1	1.5^b^	1.7^a^	< 0.1
C22:5n-6	0.5	0.5	< 0.1	0.5	0.6	< 0.1	0.5^b^	0.6^a^	< 0.1
n-6:n-3	1.22	1.20	0.1	1.19^b^	1.23^a^	0.01	1.18^b^	1.24^a^	0.01
∑CLA	13.6^a^	12.5^b^	0.5	12.9	13.3	0.5	12.3^b^	13.8^a^	0.3
c9t11CLA	9.5^a^	8.7^b^	0.4	8.9	9.2	0.4	8.4^b^	9.8^a^	0.2
t10c12CLA	4.1	3.9	0.1	3.9	4.0	0.2	4.0	4.0	0.1
∑Health claimable	31.2	31.3	0.9	30.6	34.6	0.9	28.0^b^	34.6^a^	0.3

a Abbreviations include those presented in the footnote of Tables 1 and 2.

The inclusion of lucerne (+L) resulted in the lamb meat having significantly higher concentrations of C22:0, C23:0, C24:0, C16:1n-7t, C20:4n-3, C18:2n-6, C20:4n-6, C22:4n-6 and ∑PUFA (Table 3). These individual differences contributed to lambs fed +L diets having a higher n-6 to n-3 ratio (n-6:n-3) to their counterparts fed -L diets (*P* < 0.05). The omission of lucerne (-L) resulted in lamb meat having significantly higher concentrations of C18:1n-9, C24:1n-9 and ∑MUFA (Table 3).

The LL was found to have significantly higher concentrations of C16:0, C18:0 and ∑SFA. The SM was found to have significantly higher concentrations of several individual fatty acids, as well as the sum of branch chained fatty acids (∑BCFA), CLA, ∑n-6, ∑n-3, and ∑PUFA (Table 3). The SM of the experimental lambs was found to have a significantly higher n-6:n-3 ratio (Table 3). Furthermore, the concentration of ∑EPA+DHA was higher (*P*= 0.004) in the SM (34.6 ± 0.3 mg/100 g) than in the LL (28.0 ± 0.3 mg/100 g) muscle of experimental lambs.

**Fig. 1:**
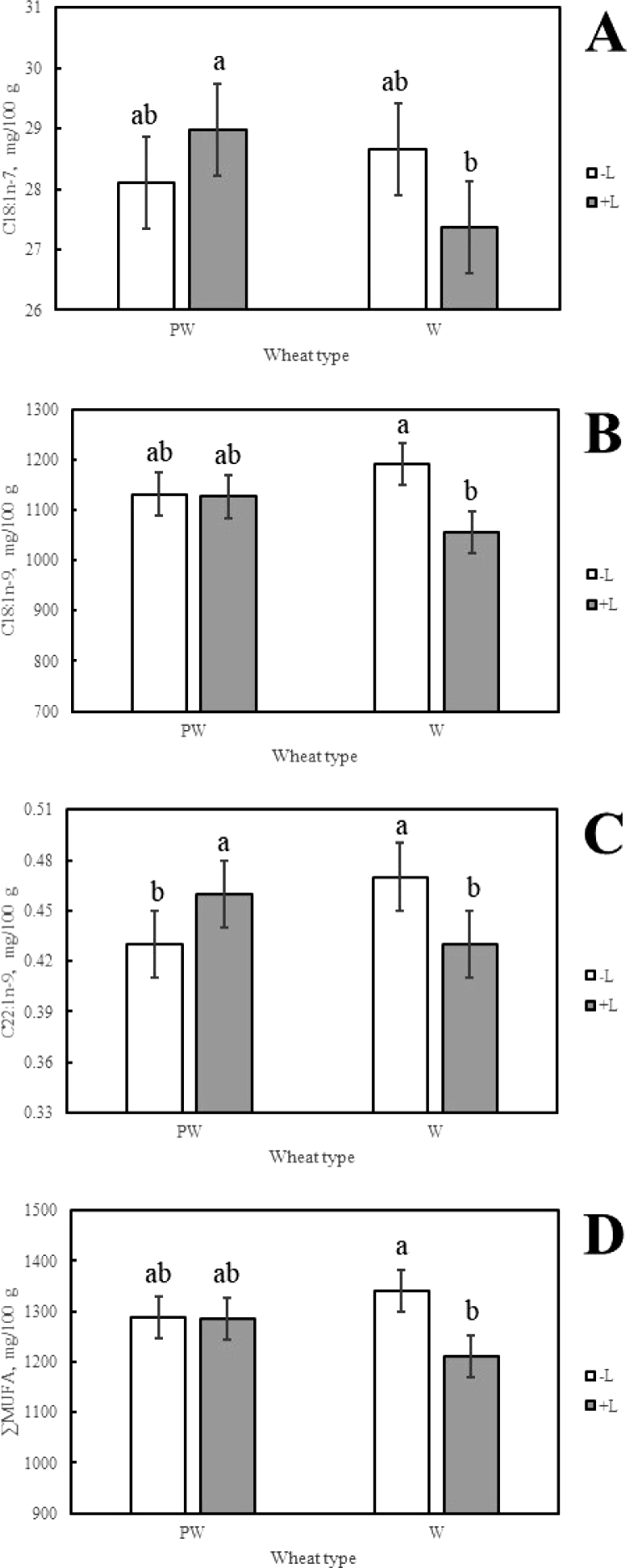
The effect of wheat type by lucerne two-way interactions on A) C18:1n-7; B) C18:1n-9; C) C22:1n-9; and D) ∑MUFA. Columns with different superscript were significantly different (*P* < 0.05). Abbreviations include plus lucerne (+L); minus lucerne (-L); perennial wheat (PW); annual wheat (W); and the sum of the monounsaturated fatty acids (∑MUFA).

The concentration of C18:1n-7 was higher in the meat of lambs fed annual wheat -L (28.7 ± 0.8 mg100 g) or perennial wheat +L (29.0 ± 0.8 mg/100 g) than was observed in the meat of lambs fed annual wheat +L (27.4 ± 0.8 mg/100 g) (Fig. 1A). The concentration of C18:1n-9 was higher in the meat of lambs fed annual wheat -L than in the meat of lambs fed this same wheat type +L (1191.5 ± 41.9 mg/100 g and 1056.2 mg/100 g, respectively) (Fig. 1B). The concentration of C22:1n-9 in the meat of lambs fed annual wheat -L (0.5 ± < 0.1 mg/100 g) or the perennial wheat +L (0.5 ± < 0.1 mg/100 g) was significantly higher than was found for lambs allocated to either of remaining dietary treatments (0.4 ± < 0.1 mg/100 g) (Fig. 1C). These individual differences in fatty acid concentration contributed to the meat of lambs fed annual wheat -L having significantly higher ∑MUFA concentrations than was observed in the meat of lambs fed annual wheat +L (1340.8 ± 39.2 mg/100 g and 1211.6 ± 39.2 mg/100 g, respectively) (Fig. 1D).

**Fig. 2:**
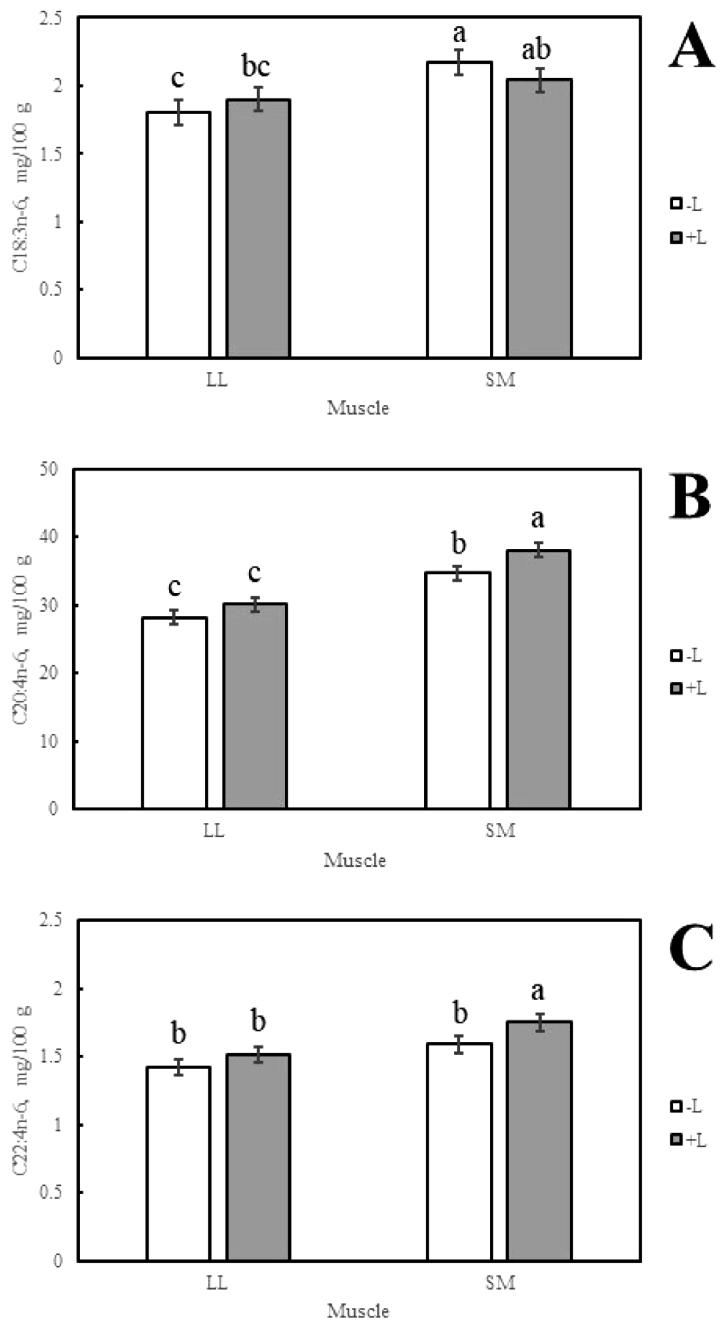
The effect of muscle by lucerne two-way interactions on (A) C18:3n-6; (B) C20:4n-6; and (C) C22:4n-6. Columns with different superscript were significantly different (*P* < 0.05). Abbreviations include plus lucerne (+L); minus lucerne (-L); m. *longissimus lumborum* (LL); and m. *semimembranosus* (SM).

The concentration of C18:3n-6 was significantly higher in the SM of lambs fed -L diets (2.2 ± 0.1 mg/100 g) than was observed in the LL of lambs fed -L or +L diets (1.8 ± 0.1 mg/100 g and 1.9 ± 0.1 mg/100 g, respectively) (Fig. 2A). The concentration of C20:4n-6 was highest in the SM of lambs fed +L diets (38.1 ± 1.0 mg/100 g), followed sequentially by the SM of lambs fed -L diets (34.7 ± 1.0 mg/100 g), and the LL of lambs fed -L and +L diets (30.2 ± 1.0 mg/100 g and 28.2 ± 1.0 mg/100 g, respectively) (*P* < 0.05, Fig. 2B). The concentration of C22:4n-6 was highest in the SM of lambs fed +L diets (1.8 ± 0.1 mg/100 g), when compared to the remaining dietary treatments (*P* < 0.05, Fig. 2C).

**Fig. 3:**
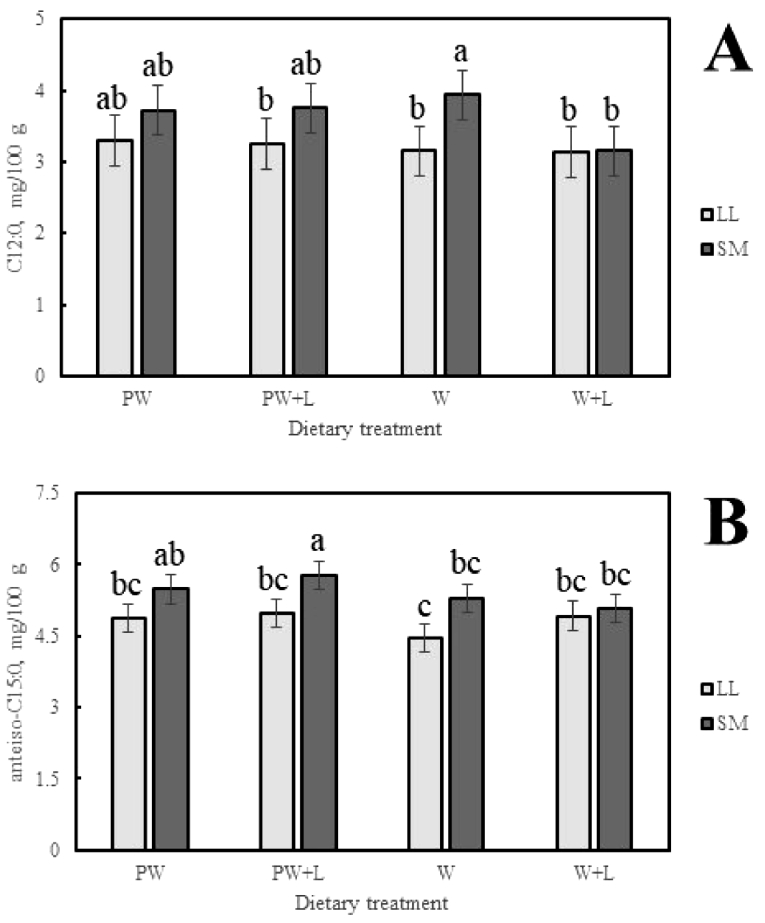
The effect of wheat type by muscle by lucerne three-way interactions on (A) C12:0; and (B) anteiso-C15:0. Columns with different superscript were significantly different (*P* < 0.05). Abbreviations include perennial wheat (PW); annual wheat (W); plus lucerne (+L); minus lucerne (-L); m. *longissimus lumborum* (LL); and m. *semimembranosus* (SM).

The concentration of C12:0 was significantly higher in the SM of lambs fed annual wheat -L than was observed in the LL of lambs fed perennial wheat +L, in the LL of lambs fed annual wheat -L or +*L*, and in the SM of lambs fed annual wheat +L and in the SM of lambs fed annual wheat (Fig. 3A). The concentration of anteiso-C15:0 was found to be highest in the SM of lambs fed perennial wheat +L (5.8 ± 0.3 mg/100 g) and lowest in the LL of lambs fed annual wheat -L (4.5 ± 0.3 mg/100 g), with the other combinations having concentrations intermediate to these results (Fig. 3B). Noting that anteiso-C15:0 is included in the calculation of ∑BCFA, a comparable trend was observed in the concentration of ∑BCFA and its concentration was also found to be highest in the SM of lambs fed perennial wheat +L (22.2 ± 0.9 mg/100 g) and lowest in the LL of lambs fed annual wheat -L (17.8 ± 0.9 mg/100 g).

### 3.2 Thiobarbituric acid reactive substances

The concentration of TBARS was significantly higher when lambs fed +L diets (0.76 ± 0.01 mg MDA/kg), when compared to their counterparts fed -L diets (0.73 ± 0.01 mg MDA/kg).

### 3.3 Vitamin e

The concentration of vitamin E was significantly higher in the meat of lambs fed +L diets (4.69 ± 0.12 mg/kg) than in the meat of lambs fed -L diets (4.27 ± 0.12 mg/kg). The concentration of vitamin E in the SM (4.84 ± 0.05 mg/kg) of experimental lambs was significantly higher than was observed in the LL (4.11 ± 0.05 mg/kg).

## 4 Discussion

Wheat type had little independent impact on the fatty acid concentration of lamb meat, including those fatty acids considered to be important for human health. Past research has demonstrated the contributions of a diet to the fatty acid concentrations of lamb meat, albeit these studies often compared the effects of forage types with considerably different nutritional and fatty acid composition (De Brito et al., 2017a; Ye et al., 2020). From the current study, it is observed that the perennial and annual wheats have a comparable fatty acid composition (Table 1). Further, the meat from lambs fed these wheats had a similar fatty acid profile to that reported by other studies of Australian lamb, wherein animals were reared under grazing management systems (Ponnampalam et al., 2014a, 2010). A point of difference, however, was that the lambs of the current study had a higher concentration of EPA and DHA to that reported in a large scale survey of Australian lamb meat (Pannier et al., 2010). Indeed, the dietary treatments of the current study resulted the concentrations of these health claimable fatty acids to be > 30 mg per 135 g serve, a value which corresponds to the FSANZ 2012 classification as a ‘source’ of omega-3 fatty acids. This result demonstrates the capacity for perennial and annual wheat to be fed to lambs so as to produce meat that is a ‘healthy’ option for consumers.

There were independent effects of lucerne on the fatty acid concentration of lamb meat, specifically to SFA and n-6 PUFA. The inclusion of lucerne in the diets of the lambs would have introduced variation to the fatty acid composition of the otherwise wheat monoculture diet (Ponnampalam et al., 2017). This may have altered the availability of these fatty acids for absorption within the small intestine of the lambs, contributing to the changes in fatty acid concentrations observed in the meat. Alternatively, lucerne is a comparatively rich source of bioactive compounds, such as saponins, which have been reported to act as antinutritional factors, impairing the activities of rumen microbiota and their synthesis of short-chain fatty acids (Sen, Makkar & Becker, 1998). These contributions to the diet could affect the passage of dietary fatty acids through the rumen (Girard et al., 2015) or reduce rumen biohydrogenation, thereby increasing the amount of fatty acids available for incorporation into the intramuscular fat (meat) of lambs. A secondary outcome from the enrichment of meat with PUFA is its increased susceptibility to peroxidation, as a result of preferential free radical autoxidation of the carbon-hydrogen bonds of long chain PUFA (Porter, Caldwell & Mills, 1995). This effect was observed in the current study as an increase to the concentration of TBARS in the meat of lambs fed +L diets. Nonetheless, comparison to thresholds presented in the literature show that all TBARS concentrations were below consumer limits for sensorial acceptance (Holman & Hopkins, 2021). Ultimately, this could be the result of the meat from experimental lambs having a higher antioxidant capacity that compensates against the peroxidation potential of PUFA.

A bioactive compound found in lucerne is vitamin E, an antioxidant associated with shelf-life extension and protection against lipid rancidification (Ponnampalam et al., 2014b). Past research has shown that the meat from lambs grazing lucerne is enriched with vitamin E (Ponnampalam, Burnett, Norng, Warner & Jacobs, 2012; Ripoll, González-Calvo, Molino, Calvo & Joy, 2013). This supports the findings of the current study, which demonstrated that lucerne was the only treatment effect to impact on the oxidative stability of lamb meat.

MUFA were the only group of fatty acids to be affected by a two-way wheat type by lucerne interaction. Feed was offered *ad libitum* and proportionally more lucerne was consumed (∼ 16%) when it was offered in addition to the perennial wheat, and compared to lambs offered annual wheat +L (Newell et al., 2020). Specifically, the provision of a biculture may have permitted lambs to select forages for palatability, rumen health, intake requirements and nutrients and minerals (Raeside et al., 2016). The mineral profile of perennial wheat is limited in its sodium content and offers potassium in concentrations that are in excess of livestock requirements (Newell & Hayes, 2017). This may have prompted compensatory intakes that would have affected the dietary intake of fatty acids, microbiota activities and the concentration of fatty acids in the meat. This premise is supported by the observed increases to MUFA in the meat of lambs fed perennial wheat +L compared to perennial wheat monocultures. Alternatively, there could be an ‘interaction’ between the phytonutrient elements of the wheat and lucerne that influences rumen passage and MUFA bioavailability. This hypothesis was, however, not tested in the current study.

Three-way interactions between wheat type, lucerne and muscle were found for only two fatty acids, with anteiso-C15:0 driving a difference in ∑BCFA. The effect may have limited practical importance when considering the potential health effects of ∑BCFA consumption (Vahmani et al., 2020). Nonetheless, the biological basis for these outcomes can be extrapolated from the knowledge that, short chain BCFA are synthesised upon the fermentation of carbohydrates by the rumen microbiota – the dietary treatments investigated in this study are shown to affect feed intake and the availability of dietary carbohydrates (Newell et al., 2020). Further, BCFA deposition is reported to vary between tissue types and muscles, for example short BCFA are more concentrated in the SM than the LL (Serra et al., 2009).

There were few two-way interactions between lucerne and muscle found to affect the fatty acid composition of lamb meat. No two-way interactions were observed between wheat type and muscle. These findings suggest a non-muscle specific response by the lambs to the dietary treatments – albeit the broader application of this observation is constrained by the current study comparing only two muscles.

There were fatty acids differences between the two muscles investigated, with the SM found to have higher concentrations of healthy fatty acids. These fatty acids included health claimable fatty acids (EPA + DHA), total branch chained fatty acids, n-3 PUFA, n-6 PUFA, and CLA fatty acids. Previous research has likewise reported similar differences between muscle fatty acid profiles (Hopkins, Holman, Fowler & Hoban, 2015). Fowler, Morris & Hopkins (2019) proposed that the muscle differences in fatty acid concentration could be the result of muscle differences in the amount of intramuscular and intermuscular fat (Holman et al., 2021), and the deposition of fatty acids within these tissues. The inclusion of intramuscular fat as a covariate in the current study would have accounted for differences in its concentration between muscles. Consequently, an alternative basis for the findings of this current study are the muscle differences in terms of the density, area and type of muscle fibres (Greenwood, Gardner & Hegarty, 2006; Ithurralde et al., 2018) which could have contributed to the observed variation in their fatty acid composition. A recent study of chevon found that Type I muscle fibres had a positive relationship to SFA and PUFA concentrations; Type I muscle fibres had a negative relationship to MUFA concentrations; and, Type IIA muscle fibres had an inverse relationship to these aforementioned fatty acids to that observed for Type I muscle fibres (Hwang, Joo, Bakhsh, Ismail & Joo, 2017). Therefore, it could be proposed a health-conscious consumer of lamb meat should prioritise meals that include cuts of the SM, over cuts of the LL. The relative absence of any diet by muscle interaction suggests this recommendation to be applicable irrespective of the lamb's basal diet.

## 5 Conclusion

The current study demonstrates the independent effect of wheat type on the concentration of fatty acids in the meat of lambs to be minimal, especially when compared to the observed effects of lucerne on the long-chain SFA and n-6 PUFA concentrations in the meat. MUFA were the only group of fatty acids affected by a wheat by lucerne interaction. There were no wheat type by muscle interactions observed to affect the concentration of fatty acids in the meat of lambs. This result somewhat reflects the minor effect of lucerne by muscle interactions on fatty acid concentrations in the meat. TBARS and vitamin E concentrations in the meat of lambs were increased with the inclusion of lucerne. Collectively, these results inform industry to the value of feeding perennial wheat as a biculture with lucerne, rather than as a cereal monoculture. Specifically, this approach enhances the nutritional value of the meat as well as improving the resilience of fatty acids to oxidation – a process that can reduce the shelf-life of lamb meat. Due to the advantages to sustainability from perennialized cereal production, the impact of other legume companion species and their potential effects on fatty acid concentrations should also be examined.

This study also found that the SM of lambs had higher concentrations of ∑BCFA, CLA, ∑n-6, ∑n-3, ∑PUFA and ∑health claimable fatty acids, when compared to the LL. These results align with past research. Collectively, therefore, these studies support the differentiation of lamb cuts when marketing product based on its relative ‘healthiness’ – when fatty acids are independently used to quantify nutritional value.

## Ethical statement

The current study adhered to the Australian Code of Practice for the Use of Animals for Scientific Purposes (National Health & Medical Research Council, 2013). Animal ethics approval was granted by the Animal Ethics Committee of the NSW Department of Primary Industries (ORA18/21/022).

## Declaration of Competing Interest

The authors declare no conflict of interest.
